# Safety and Efficacy of Long‐Term Deutetrabenazine Use in Children and Adolescents with Tics Associated with Tourette Syndrome: An Open‐Label Extension Study

**DOI:** 10.1002/mdc3.13849

**Published:** 2023-08-24

**Authors:** Joseph Jankovic, Barbara Coffey, Daniel O. Claassen, Joohi Jimenez‐Shahed, Barry J. Gertz, Elizabeth A. Garofalo, David A. Stamler, Maria Wieman, Juha‐Matti Savola, Eran Harary, Jessica Alexander, Hadas Barkay, Mark Forrest Gordon

**Affiliations:** ^1^ Parkinson's Disease Center and Movement Disorders Clinic, Department of Neurology Baylor College of Medicine Houston Texas USA; ^2^ Department of Psychiatry and Behavioral Sciences, Child and Adolescent Psychiatry University of Miami Miller School of Medicine Miami Florida USA; ^3^ Department of Neurology, Division of Behavioral and Cognitive Neurology Vanderbilt University Medical Center Nashville Tennessee USA; ^4^ Movement Disorders Neuromodulation & Brain Circuit Therapeutics, Departments of Neurology and Neurosurgery Icahn School of Medicine at Mount Sinai New York New York USA; ^5^ Nuvelution TS Pharma Inc. San Francisco California USA; ^6^ Teva Branded Pharmaceutical Products R&D, Inc. La Jolla California USA; ^7^ Teva Branded Pharmaceutical Products R&D, Inc. West Chester Pennsylvania USA; ^8^ Teva Pharmaceuticals International GmbH Basel Switzerland; ^9^ Innovative Medicines, Global Clinical Development Teva Pharmaceutical Industries Ltd. Netanya Israel; ^10^ Global Medical Affairs Teva Branded Pharmaceutical Products R&D, Inc. West Chester Pennsylvania USA; ^11^ Innovative Medicines, Global Clinical Development Teva Branded Pharmaceutical Products R&D, Inc. West Chester Pennsylvania USA

**Keywords:** deutetrabenazine, Tourette syndrome, adolescents, children, VMAT2

## Abstract

**Background:**

Tourette syndrome (TS) is a neurodevelopmental disorder characterized by motor and phonic tics.

**Objective:**

To assess the safety and efficacy of deutetrabenazine (Teva Neuroscience, Inc, Parsippany, NJ), a vesicular monoamine transporter 2 inhibitor, in children and adolescents with TS.

**Methods:**

Alternatives for Reducing Tics in TS (ARTISTS) open‐label extension (OLE) (NCT03567291) was a 54‐week, global, phase 3, open‐label extension study of deutetrabenazine (6–48 mg daily) conducted May 28, 2018 to April 3, 2020 with a 2‐week randomized withdrawal period. Participants (6–16 years of age) had TS and active tics causing distress or impairment. Safety (primary outcome) was assessed by treatment‐emergent adverse events (TEAEs) and clinical laboratory testing. Efficacy was measured by the Yale Global Tic Severity Scale‐Total Tic Score (YGTSS‐TTS).

**Results:**

The intent‐to‐treat population (228 participants; mean age, 12.0 years; 79.8% male; 86.4% white) had a median (range) duration of exposure of 28.4 (0.3–52.9) weeks. Of 227 participants in the safety analysis, 161 (70.9%) reported ≥1 TEAE (exposure‐adjusted incidence rate, 2.77/patient‐year), of which 95 (41.9%) were treatment related. The most frequently reported TEAEs were headaches, somnolence, nasopharyngitis, weight increases, and anxiety. No additional safety signals were observed. Worsening of YGTSS‐TTS after the 2‐week randomized withdrawal was not statistically significant (least squares mean difference, −0.4; *P* = 0.78). Several exploratory measures showed sustained improvement throughout the treatment periods.

**Conclusions:**

In this long‐term, open‐label trial, deutetrabenazine was well tolerated with low frequency of TEAEs. There was no significant difference in tics between treatment arms during the 2‐week randomized withdrawal period, however, descriptive statistics and comparison with baseline showed a numeric improvement in tics, quality of life, and other measures.

Tourette syndrome (TS) is a childhood‐onset neurodevelopmental disorder characterized by motor and phonic tics. Behavioral and emotional disorders, such as attention deficit hyperactivity disorder (ADHD) and obsessive‐compulsive disorder, frequently co‐occur with tics and together increase the likelihood of academic challenges, stigmatization, and poor self‐perception.^1^, ^2^, ^3^ TS has a mean age of onset of 7 years, and findings across several global regions suggest the prevalence of TS is between 0.4% and 3.8% for those between 5 and 18 years of age.^4^, ^5^, ^6^ TS affects males more frequently than females at approximately a 3:1 ratio.^6^ Tics generally cause the most impairment just before puberty and many people with TS experience partial or complete remission of tics after 18 years of age, although tics can persist through adulthood.^7^, ^8^, ^9^, ^10^ The type, intensity, and frequency of tics encompassed by TS make this disorder clinically heterogeneous.

Comprehensive behavioral intervention for tics or pharmacotherapy are usually the first treatment options for TS.^5^, ^11^ Only haloperidol, pimozide, and aripiprazole have been approved by the United States Food and Drug Administration (USFDA) for treatment of TS, but other medications are often used because of the lower probability of adverse effects such as weight gain, metabolic syndrome, and tardive dyskinesia (TD).^7^, ^12^, ^13^ The lack of therapies with favorable benefit–risk profiles justifies further pursuit of new treatments with novel mechanisms of action and fewer adverse effects.^7^ Vesicular monoamine transporter 2 (VMAT2) inhibitors reduce involuntary movements while generally exhibiting safety and tolerability and do not cause TD, unlike dopamine receptor blockers. VMAT2 inhibitors have been effective in ameliorating involuntary movements in patients with Huntington's disease (HD), TD, and other hyperkinetic movement disorders.^14^, ^15^, ^16^, ^17^

Deutetrabenazine (Teva Neuroscience, Inc, Parsippany, NJ) is a VMAT2 inhibitor approved by the USFDA in adults for treatment of TD and chorea associated with HD.^18^ A phase 1b clinical trial (NCT02674321) provided preliminary data suggesting safety and efficacy of deutetrabenazine for the treatment of tics in adolescents with TS.^19^ The Alternatives for Reducing Tics in TS (ARTISTS) program further evaluated the efficacy and safety of deutetrabenazine for treatment of tics in TS.^20^, ^21^ ARTISTS 1 (NCT03452943) and ARTISTS 2 (NCT03571256) were 12‐ and 8‐week randomized, double‐blind, placebo‐controlled, phase 3 studies of deutetrabenazine in children and adolescents with TS.^20^, ^21^ ARTISTS 1 and ARTISTS 2 did not report any additional safety signals compared with the known safety profile of deutetrabenazine,^18^ but neither showed meaningful reductions in Yale Global Tic Severity Scale‐Total Tic Score (YGTSS‐TTS) (ARTISTS 1: least squares mean difference [LSD] = −0.7 [95% confidence interval (CI) = −4.1 to 2.8], *P* = 0.69, Cohen d = −0.07; ARTISTS 2: LSD = −0.8 [95% CI = −3.9 to 2.3], *P* = 0.60, Cohen d = −0.11) compared with placebo. Of note, because ARITSTS 1 and ARTISTS 2 were 12 weeks or less, these trials were not designed to investigate efficacy and safety of long‐term deutetrabenazine treatment.

Here, we report a long‐term study, ARTISTS OLE (NCT03567291, a 56‐week, open‐label extension [OLE] study of ARTISTS 1 and ARTISTS 2), designed to evaluate the safety and tolerability of long‐term deutetrabenazine therapy for tics associated with TS in children and adolescents.

## Methods

### Study Design

ARTISTS OLE was a 56‐week phase 3, open‐label, single‐arm, 3‐part extension study for participants who completed 1 of 2 parent studies (ARTISTS 1 or ARTISTS 2).^20^, ^21^ Start of the study (day 1) for participants was the day that they enrolled, which was on or within 1 week from the week 13 or week 9 visit of ARTISTS 1 and ARTISTS 2, respectively. The initial 28‐week open‐label period comprised a 7‐week titration period followed by a maintenance period from week 8 through week 27. At week 28, participants were randomized 2:1 to continue to receive their maintenance dose of deutetrabenazine or placebo from weeks 28 to 30 (2‐week randomized withdrawal period). Following the randomized withdrawal period, doses were re‐titrated to the previous open‐label maintenance dose. Participants who received deutetrabenazine during the randomized withdrawal period continued at the same dose in a blinded manner during the withdrawal and re‐titration periods. Participants who received placebo during the randomized withdrawal period underwent blinded re‐titration back to their maintenance dose from the start of week 31 to week 34. All participants were receiving their maintenance dose of deutetrabenazine by the start of week 34 and continued open‐label treatment up to week 54. Participants and investigators remained blinded to treatment assignment during the study. Sponsor, development partner clinical personnel, and all vendors involved were also blinded to the study drug identity until the database was locked for analysis. The final period of the study was a 2‐week follow‐up period, *off* medication. During the follow‐up period, assessments were performed at week 55 (1 week after treatment completion) and adverse events were collected via telephone 2 weeks after the last dose of deutetrabenazine.

### Participants

Participants were eligible for ARTISTS OLE after a 1‐week washout following completion of 1 of the double‐blind, placebo‐controlled parent studies. The key inclusion criteria for the ARTISTS 1 and ARTISTS 2 parent studies were 6 to 16 years of age (inclusive), at least 20 kg body weight at baseline, diagnosis of TS per Diagnostic and Statistical Manual of Mental Disorders, 5th Edition criteria, active tics causing distress or impairment, and an YGTSS‐TTS of at least 20. Key exclusion criteria included stereotypy associated with autism spectrum disorder or diagnosis of bipolar disorder, schizophrenia, or another psychotic disorder.^20^, ^21^

### Interventions

Participants received deutetrabenazine twice daily (6–48 mg/day, determined by body weight and cytochrome P450 2D6 [CYP2D6] impairment status on day 1) for up to 54 weeks (participants on the 6‐mg dose received once‐daily dosing), with an initial 7 week titration period. During the titration period, dose adjustments could be made no more than once every 5 days and only in 6‐mg increments; dose adjustments were not allowed during the maintenance periods.

### Assessments

Safety was assessed by evaluating treatment‐emergent adverse events (TEAEs), clinical laboratory test results, vital sign measurements, electrocardiogram (ECG) findings, physical examination findings (including body weight and height measurements), concomitant medication usage, neurological examination, Columbia‐Suicide Severity Rating Scale, and Children's Depression Inventory 2nd Edition (CDI 2). The key secondary endpoint was the change in the YGTSS‐TTS from week 28 to week 30. The YGTSS is a semi‐structured clinician rating instrument that evaluates the number, frequency, intensity, complexity, and interference of motor and phonic tics.^22^ Exploratory endpoints included change in the YGTSS‐TTS from baseline and the change in the Tourette Syndrome‐Clinical Global Impression scale (TS‐CGI) from baseline. TS‐CGI is a 7‐point Likert scale that assesses the impact of tics on a patient's quality of life. Additional endpoints included Tourette Syndrome‐Patient Global Impression Impact (TS‐PGII), Child and Adolescent Gilles de la Tourette Syndrome‐Quality of Life (C&A‐GTS‐QoL), Children's Yale‐Brown Obsessive‐Compulsive Scale (CY‐BOCS), Tourette Syndrome‐Patient Global Impression of Severity (TS‐PGIS), YGTSS global severity score (YGTSS GSS), tic‐free interval, and visual analogue scale in the C&A‐GTS‐QoL.

### Statistical Analysis

The intent‐to‐treat (ITT) analysis set includes all participants enrolled in the study, regardless of whether they received study medication. The safety analysis set includes all participants who received at least one dose of deutetrabenazine. The responder‐randomized, withdrawal‐modified ITT population includes all participants who completed the initial 28‐week open‐label period, were randomized, received study medication during the randomized withdrawal period, had YGTSS‐TTS results at both the week‐28 and week‐30 visits, and had a ≥25% reduction in the YGTSS‐TTS from baseline in the parent study to week 28.^23^ All data were processed and summarized using SAS Version 9.4 (SAS Institute, Carey, NC). No inferential statistics were determined for safety measures and exploratory endpoints from the initial open‐label period or the re‐titration, open‐label maintenance, and follow‐up period; however, inferential statistics were applied to efficacy endpoints from the randomized withdrawal period. Statistical analysis of YGTSS‐TTS was performed with an analysis of covariance (ANCOVA) model that included fixed effects for treatment group where the randomized withdrawal baseline and age group at baseline were covariates.

### Ethics Committee and Consent

The protocol was submitted to the appropriate Independent Ethics Committee or Institutional Review Board according to local regulations. Signed and dated informed consent from the parent/legally acceptable representative and a signed and dated assent, if necessary, was obtained for each participant.

## Results

### Patient Characteristics

**FIG. 1:**
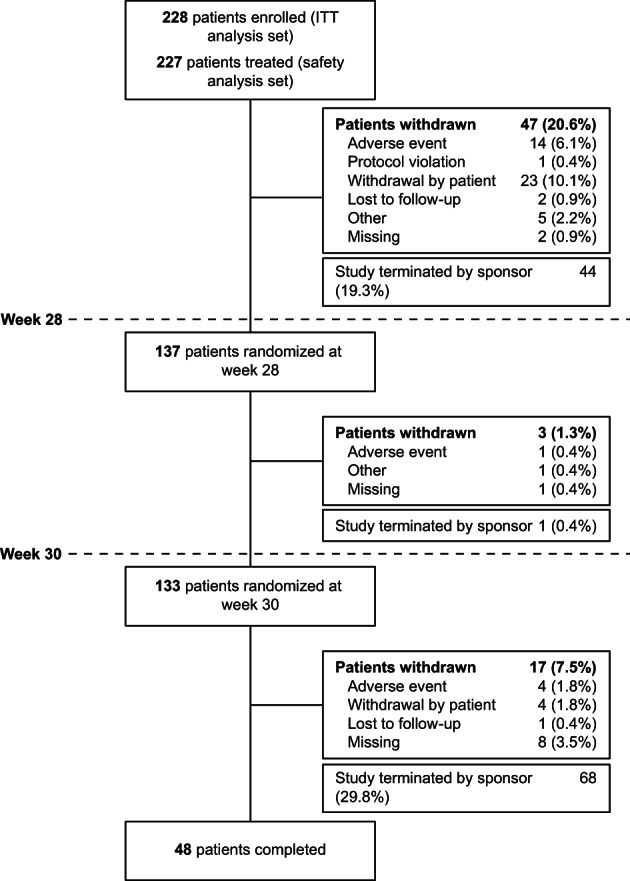
Patient disposition. Proportions are based on the number of patients in the intent‐to‐treat (ITT) analysis set.

Of the 228 participants who were enrolled, all were included in the ITT population with a mean age of 12 years (range, 6–17 years) (Table 1). The mean (standard deviation [SD]) baseline YGTSS‐TTS was 27.5 (9.24). The study was terminated early following evaluation of the clinical program based on the results of the ARTISTS 1 and ARTISTS 2 parent studies,^20^, ^21^ which did not meet their primary efficacy endpoints, and not because of any safety concerns. At the time of study termination, recruitment had been completed; 137 (60.1%) participants completed the initial open‐label period, 48 (21.1%) participants completed the study, and participants who were ongoing were discontinued (Fig. 1). Although there is a low proportion of participants that completed the study, it is important to note that a large portion were withdrawn because of study termination: 44 (19.3%) before week 28, 1 (0.4%) during weeks 28 to 30, and 68 (29.8%) after week 30. Of 227 patients that received deutetrabenazine, the mean (SD) duration of exposure was 27.7 (14.0) weeks.

**TABLE 1:** Demographic and baseline characteristics

Demographic variables	All patients, N = 228
Age, years^a^	
Mean (SD)	12.0 (2.59)
Median (range)	12.0 (6–17)
Extension study age group, years, n (%)^a^	
6–11	95 (41.7)
12–18	133 (58.3)
Parent study age group, years, n (%)^b^	
6–11	106 (46.5)
12–18	122 (53.5)
Sex, n (%)^c^	
Male	182 (79.8)
Female	45 (19.7)
Race, n (%)^d^, ^e^	
Asian	7 (3.1)
Black	4 (1.8)
Multiple	4 (1.8)
Native American	5 (2.2)
Other	11 (4.8)
White	197 (86.4)
Not Hispanic or Latino, n (%)^d^	186 (81.6)
Time since Tourette syndrome diagnosis, years^c^	
Mean (SD)	3.33 (2.792)
Median (range)	2.44 (0.2–14.4)
Weight, kg^e^	
Mean (SD)	53.17 (21.393)
Median (range)	49.80 (20.8–157.0)
Weight group, kg, n (%)^e^	
20 to <30	23 (10.1)
30 to <40	46 (20.2)
≥40	155 (68.0)
BMI, kg/m^2^ ^f^	
Mean (SD)	21.46 (5.638)
Median (range)	20.27 (12.1–51.0)
BMI percentile, kg/m^2^ ^g^	
Mean (SD)	68.27 (32.488)
Median (range)	80.78 (0.0–100.0)
BMI category, n (%)^g^	
Underweight	9 (3.9)
Normal	110 (48.2)
Overweight	33 (14.5)
Obese	68 (29.8)
Strong CYP2D6 inhibitor use, n (%)	21 (9.2)
Poor CYP2D6 metabolizer, n (%)	13 (5.7)
CYP2D6 impaired, n (%)	33 (14.5)
YGTSS‐TTS^h^	
Mean (SD)	27.5 (9.24)
Median (range)	29.0 (3–49)

*Note*: Intent‐to‐treat analysis set used for analysis.

Abbreviations: SD, standard deviation; BMI, body mass index; CYP2D6, cytochrome P450 2D6; YGTSS‐TTS, Yale Global Tic Severity Scale‐Total Tic Score.

^a^ Age at start of the open‐label extension.

^b^ Age at the parent study baseline.

^c^ Data were missing for 1 patient.

^d^ Race information was collected using predefined options in the case report form.

^e^ Data were missing for 4 patients.

^f^ Data were missing for 6 patients.

^g^ Data were missing for 8 patients.

^h^ Data were missing for 3 patients.

### Safety Assessment

**FIG. 2:**
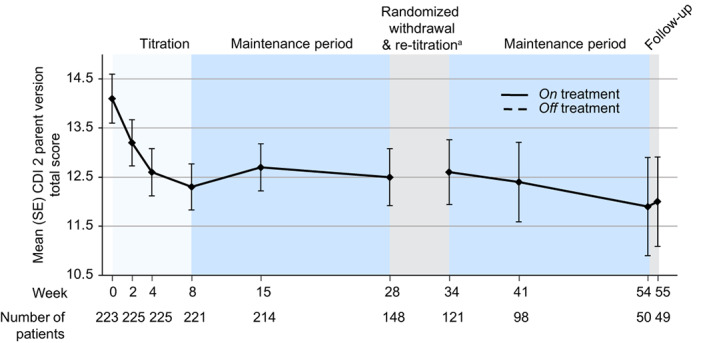
Children's Depression Inventory, Second Edition (CDI 2) parent version. Baseline (week 0) is defined as the last measurement on or before the first dose of the open‐label study medication. ^a^Participants were randomized 2:1 to undergo blinded withdrawal from medication from weeks 28 to 30. Mean (standard error [SE]) for CDI 2 at week 28 was 11.7 (0.81) and 13.6 (1.05) and at week 30 was 11.7 (0.83) and 14.2 (1.16) for the deutetrabenazine and placebo treatment groups, respectively, for the randomized withdrawal safety population.

In total 227 participants were in the safety population, and 161 (70.9%) participants reported at least 1 TEAE, equating to an exposure‐adjusted incidence rate (EAIR) of 2.77/patient‐year (Table 2). Adverse events considered by the investigator to be treatment related were reported for 95 (41.9%, EAIR of 1.10/patient‐year) participants. There were no deaths reported in the study, but six (2.6%, EAIR of 0.05/patient‐year) participants reported severe TEAEs (Table 2). Two participants (0.9%, EAIR of 0.02/patient‐year) had a serious adverse event (Table 2), and one (0.4%, EAIR of 0.01/patient‐year) participant had a serious adverse event considered by the investigator to be treatment related (self‐injurious ideation during the initial 28‐week open‐label period of the study, moderate with reasonable possibility of being related to study drug). During the study, 14 (6.2%, EAIR of 0.11/patient‐year) participants discontinued deutetrabenazine because of adverse events, 12 (5.3%, EAIR of 0.10/patient‐year) participants had an adverse event leading to dose interruption, and 17 (7.5%, EAIR of 0.14/patient‐year) participants had an adverse event leading to a dose reduction (Table 2). Adverse events that led to study drug discontinuation in ≥2 participants included depressed mood (n = 2, 0.9%, EAIR of 0.02/patient‐year) and increased weight (n = 2, 0.9%, EAIR of 0.02/patient‐year) (Table 3). TEAEs were reported for similar proportions of participants in both the 6 to 11 and the 12 to 16 age groups (73.6% and 68.6%, respectively). The most frequently reported adverse event was headaches (30 [13.2%] participants, EAIR of 0.27/patient‐year), whereas other frequently observed adverse events included somnolence, nasopharyngitis, weight increases, and anxiety (Table 2). Few participants increased, decreased, or stopped their ADHD or antidepressant medication, and no participant switched these medications (Table 4). For the depression assessment CDI 2 parent version, mean total and subscale raw scores showed a small decreasing trend over time; however, these results should be considered in light of the substantial interpatient variability (Fig. 2). No significant findings were observed regarding laboratory, vital signs, ECG, or physical examinations. Safety scales indicated that there were no clinically significant increases in symptoms of ADHD, depression, or suicidality.

**TABLE 2:** Overview of adverse events for parts 1–3

Adverse event category	All patients, N = 227	
	n (%)	EAIR^a^
Patients with any TEAE	161 (70.9)	2.77
Patients with any treatment‐related TEAE^b^	95 (41.9)	1.10
Patients with any severe TEAE	6 (2.6)	0.05
Patients with any serious TEAE^c^	2 (0.9)	0.02
Patients with any serious treatment‐related TEAE	1 (0.4)	0.01
Patients with any TEAE leading to study drug discontinuation	14 (6.2)	0.11
Patients with any TEAE leading to dose interruption	12 (5.3)	0.10
Patients with any TEAE leading to dose reduction	17 (7.5)	0.14
Patients with any TEAE leading to death	0	0
Most frequently reported TEAEs (≥4%)^b^		
Headache	30 (13.2)	0.27
Somnolence	28 (12.3)	0.25
Nasopharyngitis	23 (10.1)	0.20
Weight increased	22 (9.7)	0.19
Anxiety	17 (7.5)	0.14
Tic	15 (6.6)	0.12
Vomiting	15 (6.6)	0.13
Pyrexia	13 (5.7)	0.11
Fatigue	11 (4.8)	0.09
Upper respiratory tract infection	11 (4.8)	0.09
Influenza	10 (4.4)	0.08
Nausea	10 (4.4)	0.08
Diarrhea	9 (4.0)	0.07

*Note*: Safety analysis set used for analysis.

Abbreviations: EAIR, exposure‐adjusted incidence rate; TEAE, treatment‐emergent adverse event; MedDRA, Medical Dictionary for Regulatory Activities; AE, adverse event.

^a^ EAIR is calculated as the number of patients with an adverse event divided by patient‐years of treatment. Patients with an TEAE contribute to the treatment exposure up to the day of their first contributing TEAE, and patients without a TEAE contribute their entire treatment duration.

^b^ Adverse events are classified by system organ class and preferred term using MedDRA version 22.1. Patients who experience the same coded event more than once are counted only one time per preferred term and one time per system organ class.

^c^ One patient had a serious AE 12 days after the last dose of treatment, and therefore, was not included in the overall AE data.

**TABLE 3:** Adverse events leading to study drug discontinuation

MedDRA System Organ Class MedDRA preferred term^a^	All patients, N = 227	
	n (%)	EAIR^b^
Patients with at least 1 TEAE leading to study drug discontinuation	14 (6.2)	0.11
Psychiatric disorders	6 (2.6)	0.05
Depressed mood	2 (0.9)	0.02
Aggression	1 (0.4)	0.01
Anxiety	1 (0.4)	0.01
Depression	1 (0.4)	0.01
Self‐injurious ideation	1 (0.4)	0.01
Tic	1 (0.4)	0.01
Investigations	3 (1.3)	0.02
Weight increased	2 (0.9)	0.02
Hepatic enzyme increased	1 (0.4)	0.01
Gastrointestinal disorders	2 (0.9)	0.02
Abdominal discomfort	1 (0.4)	0.01
Abdominal pain	1 (0.4)	0.01
Gastroesophageal reflux disease	1 (0.4)	0.01
Nervous system disorders	2 (0.9)	0.02
Restless legs syndrome	1 (0.4)	0.01
Seizure‐like phenomena	1 (0.4)	0.01
Skin and subcutaneous disorders	1 (0.4)	0.01
Rash	1 (0.4)	0.01

*Note*: Safety analysis set used for analysis.

Abbreviations: EAIR, exposure‐adjusted incidence rate; MedDRA, Medical Dictionary for Regulatory Activities; TEAE, treatment‐emergent adverse event.

^a^ Adverse events are classified by system organ class and preferred term using MedDRA version 22.1. Patients who experience the same coded event more than once are counted only one time per preferred term and one time per system organ class.

^b^ EAIR is calculated as the number of patients with an adverse event divided by patient years of treatment. Patients with an adverse event contribute to the treatment exposure up to the day of their first contributing adverse event, and patients without an adverse event contribute their entire treatment duration.

**TABLE 4:** ADHD and antidepressant medication changes

Medication category	Post‐baseline change	All patients, N = 227 baseline use	
		No, n (%)	Yes, n (%)
ADHD	M^a^	176	51
	New	8 (4.5)	0
	Increase	0	7 (13.7)
	Decrease/stop	0	3 (5.9)
	Switch	0	0
Antidepressant	M^a^	210	17
	New	2 (1.0)	0
	Increase	0	1 (5.9)
	Decrease/stop	0	2 (11.8)
	Switch	0	0

*Note*: Safety analysis set used for analysis.

^a^ Proportions were based on M, the number of patients with ADHD/antidepressant medication use at baseline. Patients who had no ADHD/antidepressant medication use at baseline or post‐baseline during parts 1 or 3 were not included in this analysis, but were not excluded in percentage computation.

Abbreviations: ADHD, attention deficient hyperactivity disorder.

### Efficacy Assessment

During the 2‐week randomized withdrawal period (weeks 28–30), a numeric worsening was observed in both groups, as follows: at week 28 (start of randomized withdrawal), mean (SD) YGTSS‐TTS was 14.8 (6.57) and 14.3 (7.52) for the deutetrabenazine and placebo treatment groups, respectively; at week 30, there were mean (standard error [SE]) increases (worsening) in the YGTSS‐TTS from the start of the randomized withdrawal period (week 28) of 1.5 (0.78) and 1.9 (1.44) for the deutetrabenazine and placebo treatment groups, respectively. The results were not clinically meaningful or statistically significant (LSD = −0.4; *P* = 0.78). A ≥20% increase in YGTSS‐TTS during the randomized withdrawal period occurred in 17 (32.1%) participants in the deutetrabenazine group and 10 (40%) in the placebo group.

**FIG. 3:**
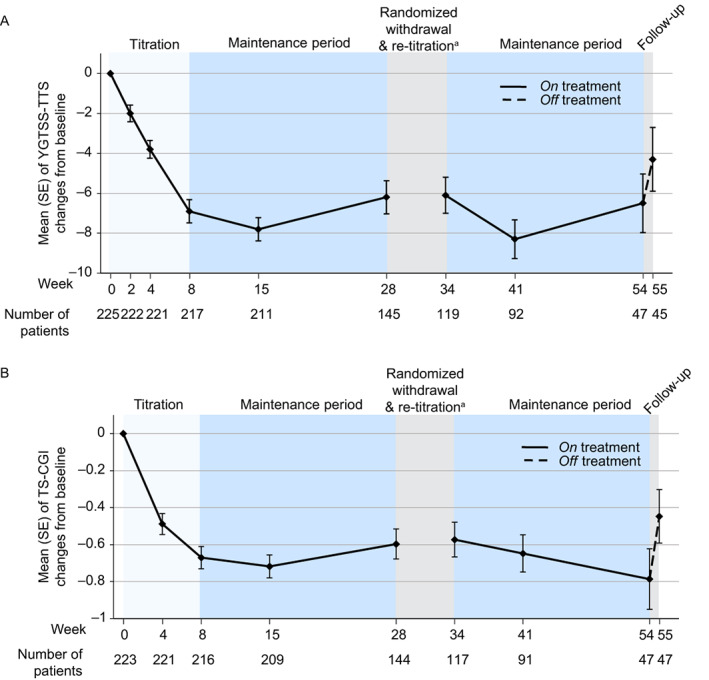
Tic severity and impact of tics. Baseline (week 0) is defined as the last measurement on or before the first dose of the open‐label study medication. Intent‐to‐treat analysis set used for analysis. ^a^Participants were randomized 2:1 to undergo blinded withdrawal from medication from weeks 28 to 30. Mean (standard error [SE]) change from week 28 for Yale Global Tic Severity Scale‐Total Tic Score (YGTSS‐TTS) at week 30 was 1.5 (0.78) and 1.9 (1.44) for the deutetrabenazine and placebo treatment groups, respectively, for the responder‐randomized withdrawal‐modified intent‐to‐treat population. Tourette Syndrome‐Clinical Global Impression (TS‐CGI) was not assessed at week 30, during the randomized withdrawal and re‐titration period.

**FIG. 4:**
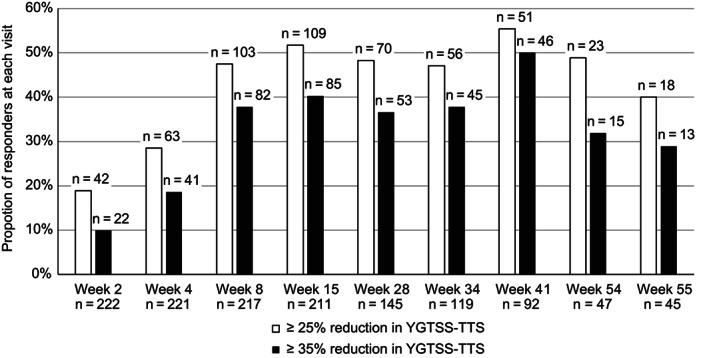
Responder rates at each visit. TGTSS‐TTS, Yale Global Tic Severity Scale‐Total Tic Score.

In the initial 28‐week open‐label period and the re‐titration, open‐label maintenance, and follow‐up periods combined, descriptive statistics revealed numeric reductions in tics from baseline to the end of treatment; symptoms worsened after the end of treatment. At baseline, the mean (SD) YGTSS‐TTS was 27.5 (9.24), and a sustained decrease (indicating improvement) in the mean was observed at weeks 8, 15, 28, 41, and 54, respectively, with a slight increase (indicating worsening) at follow‐up week 55 after a 1‐week washout from active treatment. Mean (SE) reductions in YGTSS‐TTS at each on‐treatment visit were similar, with a range of −6.2 (0.83) to −8.3 (0.97) (Fig. 3A). At week 54 (end of treatment visit), a mean (SE) reduction of −6.5 (1.47) was observed. At week 55 (follow‐up visit), YGTSS‐TTS was below baseline but elevated compared with on‐treatment visits. Responder rates for ≥25% and ≥35% reduction (improvement) in YGTSS‐TTS at week 54 were 23 (48.9%) and 15 (31.9%), respectively (Fig. 4).

Quality of life related to tics was assessed and showed improvement that was sustained through the treatment period but worsened 1 week after treatment ended. At baseline, the mean (SD) TS‐CGI was 4.0 (0.85) and through the initial 28‐week open‐label period and the re‐titration, open‐label maintenance and follow‐up period there was a sustained decrease at weeks 8, 15, 28, 41, and 54 with a slight increase at the week 55 follow‐up. At week 8, the mean (SE) change from baseline was −0.7 (0.06), which was sustained through week 54, and at week 55 (follow‐up visit) mean TS‐CGI scores were worse than at any on‐treatment visit (Fig. 3B).

Descriptive statistics of several exploratory measures showed similar patterns of sustained improvement throughout the treatment periods. Impact of tics on TS‐PGII was rated as “somewhat” to “a lot” (mean, 2.7) at baseline, and at week 8 there was a mean (SE) change of −0.5 (0.07), indicating improvement, which was sustained through week 54. At baseline, mean (SD) of the C&A‐GTS‐QoL activities of daily living subscale score was 25.0 (19.52) and there was a mean (SE) change of −4.4 (1.48) at week 28 and −2.7 (2.10) at week 54, suggesting benefit of treatment. Obsessive‐compulsive behavior was measured by CY‐BOCS; mean (SD) total score was 3.0 (6.16) and improvements were indicated by mean (SE) changes from baseline of −0.7 (0.38) at week 28 and −1.7 (0.59) at week 54. Impact of tics on TS‐PGIS score, YGTSS impairment score, YGTSS GSS, and tic‐free interval improved by weeks 2 to 4, and improvements were sustained throughout the treatment period. The visual analogue scale in the C&A‐GTS‐QoL indicated a nominal improvement by week 28, which was sustained throughout the treatment period.

## Discussion

This long‐term, open‐label study demonstrates that use of deutetrabenazine, a VMAT2 inhibitor, in children and adolescents with TS has a low frequency of TEAEs, and suggests that in this pediatric population, deutetrabenazine does not produce any new safety signals beyond the known safety profile.^18^, ^20^, ^21^ Although there was no significant difference in tics between the active and placebo arms during the 2‐week randomized withdrawal period, descriptive statistics and comparison with baseline showed a numeric reduction in tics and improvements in quality of life and other secondary measures through the end of treatment, followed by a numeric increase (worsening) at follow‐up 1 week after treatment was withdrawn compared with the last on‐treatment visit.

This open‐label trial was terminated early, because of the parent studies, ARTISTS 1 and ARTISTS 2,^20^, ^21^ not meeting their primary endpoints. There were no concerns in safety outcomes. Deutetrabenazine was well tolerated in this pediatric population, with low rates of adverse events leading to study drug discontinuation, study drug interruptions, and dose reductions. The most frequently reported adverse events were similar to those reported in the adult population of patients with TD or chorea in HD who are treated with deutetrabenazine. There were no clinically meaningful changes in depression or suicidality measures with prolonged treatment with deutetrabenazine. Similar to these findings and previous literature of deutetrabenazine for chorea in HD,^16^ another VMAT2 inhibitor, tetrabenazine, was not associated with an increased incidence of depression or suicidality in a population of patients with HD.^24^

In this study of children and adolescents with tics associated with TS, open‐label treatment with deutetrabenazine produced no significant difference between the active and placebo arms during the randomized withdrawal period lasting 2 weeks. It is possible, however, that the 2‐week withdrawal period was too short to observe any statistical difference or clinically meaningful increase in tics. However, descriptive statistics and comparison with baseline showed a numeric reduction in tics and improvements in quality of life and other secondary measures through the end of treatment, followed by a numeric increase (worsening) at follow‐up 1 week after treatment was withdrawn compared with the last on‐treatment visit. At follow‐up 1 week after treatment was withdrawn, no placebo‐controlled statistical analysis was performed, and the likelihood of a placebo effect is high. Therefore, worsening during the washout period may be biased.

The parent studies (ARTISTS 1 and ARTISTS 2) did not meet their primary endpoints, possibly because of marked heterogeneity in the patient population and subtherapeutic dosing.^18^, ^19^ Another VMAT2 inhibitor, valbenazine, failed to show significant differences from placebo in YGTSS‐TTS in two phase 2, placebo‐controlled trials in the pediatric population with TS.^25^ However, published open‐label^19^ and real‐world experiences^15^, ^17^ with deutetrabenazine and other VMAT2 inhibitors indicate that this class of anti‐dopaminergic agents is well tolerated and may show efficacy in the treatment of tics associated with TS when appropriately dosed.^17^ Because the maximum dose for deutetrabenazine, approved by the USFDA, is 48 mg/day, this study was designed to not exceed this dose. However, in real‐world experience, dosages as high as 96 mg/day have been required for optimal treatment response.^15^, ^17^ Our study confirms that deutetrabenazine does not pose additional tolerability concerns in the pediatric and adolescent population.^20^, ^21^

### Limitations

Limitations of the study include the open‐label design and the early termination. As an extension study that invited participants who had already received deutetrabenazine, there may be bias toward participants with a favorable deutetrabenazine response. Higher variability in results was observed by week 54 for the majority of endpoints, which can be attributed to the smaller number of participants who completed the end of treatment visit before study termination. The randomized withdrawal period was 2 weeks, and this duration may not have been sufficient to evaluate persistence of effect. Additionally, effects from the withdrawal period may have been masked by a strong placebo effect, which has been documented for YGTSS‐TTS assessments in patients with TS.^25^ Although the overall overweight and obesity observed in the general population of 2‐ to 18‐year‐olds is 31.2%,^26^ this dataset included 44.3% overweight and obese participants. The greater number of participants classified as overweight or obese may limit the generalizability of these results. The participant population in this study was mostly White, non‐Hispanic male children and adolescents, which reduces generalizability across populations with differing demographic distributions (ie, sex, race, and ethnicity). This study and the parent studies focused on children and adolescents, but the applicability of deutetrabenazine treatment in the adult population with TS requires additional studies.

### Conclusions

In this long‐term, open‐label extension study in pediatric patients with TS, deutetrabenazine was generally well tolerated with a low frequency of adverse events, which is consistent with the known safety profile of deutetrabenazine in studies conducted in adults. Although there was no significant difference in tics between the active and placebo arms during the 2‐week randomized withdrawal period, descriptive statistics and comparison with baseline showed a numeric reduction in tics and improvements in quality of life and other outcome measures, followed by a worsening at follow‐up 1 week after treatment was withdrawn.

## Author Roles

(1) Research project: A. Conception, B. Organization, C. Execution; (2) Statistical Analysis: A. Design, B. Execution, C. Review and Critique; (3) Manuscript Preparation: A. Writing of the First Draft, B. Review and Critique.

J.J.: 1A, 1C, 2A, 2C, 3B

B.C.: 1A, 1C, 2A, 2C, 3B

D.O.C.: 1A, 2A, 2C, 3A

J.J.S.: 2B, 2C, 3B

B.J.G.: 2C, 3B

E.A.G.: 1A, 1C, 2A, 2C, 3B

D.A.S.: 1A, 1C, 2A, 3A, 3B

M.W.: 1A, 2A, 3A, 3B

J.M.S.: 1A, 2A, 3A, 3B

E.H.: 1C, 2C, 3B

J.A.: 1A, 2A, 2C, 3A, 3B

H.B.: 2B, 2C, 3A, 3B

M.F.G.: 1B, 2B, 2C, 3A, 3B

## Disclosures

**Ethical Compliance Statement:** Protocols were approved by the appropriate national and local authorities and independent ethics committees or institutional review boards at each study site before study initiation. Protocol amendments were also submitted. The study was conducted in full accordance with the International Council for Harmonization Good Clinical Practice Consolidated Guideline E6 and any applicable national and local laws and regulations. A personally signed and dated informed consent form was obtained from the parent/legally acceptable representative, and a signed and dated assent, depending on the child's age, as appropriate, was obtained from each patient (if the patient was able) before any study‐specific procedures or assessments were performed, and after the aims, methods, anticipated benefits, and potential hazards were explained. We confirm that we have read the Journal's position on issues involved in ethical publication and affirm that this work is consistent with those guidelines.

**Funding Sources and Conflicts of Interest:** This study was funded by Teva Branded Pharmaceutical Products R&D, Inc. The funders were involved in the design and conduct of the study; collection, management, analysis, and interpretation of the data; preparation, review, or approval of the manuscript; and decision to submit the manuscript for publication. D.A.S., M.W., and J.A. were employees of the funder at the time of this research. M.F.G. is an employee of the funder. J.M.S. was an employee of Teva Pharmaceuticals International GmbH at the time of this research, and E.H. and H.B. are employees of Teva Pharmaceutical Industries Ltd.

**Financial Disclosures for Previous 12 Months:** J.J. reported receiving grants or contracts from AbbVie, CHDI Foundation, Dystonia Coalition, Emalex Biosciences, Medtronic Neuromodulation, The Michael J. Fox Foundation for Parkinson Research, Parkinson's Foundation, Revance Therapeutics, and Teva Pharmaceutical Industries, royalties or licenses from Cambridge, Elsevier, Medlink: Neurology, Lippincott Williams and Wilkins, UpToDate, and Wiley‐Blackwell; and consulting fees from AbbVie, Eon BioPharma, Neurocrine, Revance Therapeutics, and Teva Pharmaceutical Industries. B.C. reported receiving grants, honoraria or consulting fees from the American Academy of Child and Adolescent Psychiatry; Emalex; Harvard Medical School /Psychiatry Academy; The National Institute of Mental Health; Partners Healthcare; Skyland Trail; Neurocrine; Teva/Nuvelution; and Tourette Association of America‐CDC Partnership. D.O.C. reported receiving grants or contracts from AbbVie, Acadia Pharmaceuticals, Alerity Therapeutics, Annexon, Neurocrine, Genentech‐Roche, Novartis, Prilenia, Spark Therapeutics, and uniQure; consulting fees from Annexon, Spark Therapeutics, Alterity Therapeutics, Novartis, and Teva Neuroscience; data safety monitoring board or advisory board duties from Photopharmics; and leadership or fiduciary role in MSA Coalition and Huntington Study Group. J.J.S. reported receiving consulting fees from Teva Branded Pharmaceutical Products R&D, Nuvelution, Bracket/Signant Health, St. Jude Medical, Revance Therapeutics, Amneal, Impel Pharmaceuticals, Medtronic, and AbbVie. She has received research fees from Amneal. She serves on the data safety monitoring board for BlueRock Therapeutics, and has advisory board duties for Photopharmics. B.J.G. was an employee of Nuvelution and Blackstone Life Sciences. E.A.G. reported receiving consulting fees from Nuvelution and TS Pharma Inc.
